# Community dynamics during de novo colonization of the nascent peri-implant sulcus

**DOI:** 10.21203/rs.3.rs-5390824/v1

**Published:** 2024-12-13

**Authors:** Purnima Kumar, Tamires Dutra, Nicolas Robitaille, Khaled alTabtbaei, Shareef Dabdoub

**Affiliations:** University of Michigan School of Dentistry; University of Michigan School of Dentistry; Private practice; University of Alberta; University of Iowa

## Abstract

Dental implants have restored chewing function to over 100,000,000 individuals, yet almost 1,000,000 implants fail each year due to peri-implantitis, a disease triggered by peri-implant microbial dysbiosis. Our ability to prevent and treat peri-implantitis is hampered by a paucity of knowledge of how these biomes are acquired and the factors that engender normobiosis. Therefore, we combined a 3-month interventional study of 15 systemically and periodontally healthy adults with whole genome sequencing, fine-scale enumeration and graph theoretics to interrogate colonization dynamics in the pristine periimplant sulcus. We discovered that colonization trajectories of implants differ substantially from adjoining teeth in acquisition of new members and development of functional synergies. Source-tracking algorithms revealed that this niche is initially seeded by bacteria trapped within the coverscrew chamber during implant placement. These pioneer species stably colonize the microbiome and exert a sustained influence on the ecosystem by serving as anchors of influential hubs and by providing functions that enable cell replication and biofilm maturation. Unlike the periodontal microbiome, recruitment of new members to the peri-implant community occurs on nepotistic principles. Maturation is accompanied by a progressive increase in anaerobiosis, however, the predominant functionalities are oxygen-dependent over the 12-weeks. The peri-implant community is easily perturbed following crown placement, but demonstrates remarkable resilience; returning to pre-perturbation states within three weeks. This study highlights important differences in the development of the periodontal and peri-implant ecosystems, and signposts the importance of placing implants in periodontally healthy individuals or following the successful resolution of periodontal disease.

## INTRODUCTION

Nearly 60% of Americans over 50 years have less than 21 teeth; the minimum required for optimal function(1). Partial or complete edentulism places an enormous burden on an individual’s physical (oral and general health) and psychological well-being(2,3), as well as financial cost(3). In 1978, the titanium root-form implant was introduced(4); and by the 1990s, two novel biofilm-induced inflammatory diseases had been documented: peri-implant mucositis and peri-implantitis(5). Peri-implantitis occurs in 1/10^th^ of all implants, and peri-implant mucositis in 60% of implants(6).The onset and course of peri-implantitis is highly unpredictable; and an implant can be lost within a few months of diagnosis, leading to large soft-tissue and bone deficits that require significant reconstruction(7). Evidence implicates history of periodontal disease, poor plaque control and lack of compliance with maintenance therapy in increasing the risk for peri-implantitis; and the common thread linking these factors is that they all contribute to creating and perpetuating dysbiotic implant microbiomes (8).

In order to elucidate the role of microbial dysbiosis in the onset and progression of peri-implant diseases, it is important to define the characteristics of a normobiotic community. While natural teeth have evolved to recognize microbial patterns and establish a homeostatic relationship with the resident community, implants lack a similar evolutionary heritage. Indeed, it is now established that the topography and surface characteristics of dental and other biomedical implants alter the natural environment, and that the peri-implant microbiome differs substantially from subgingival microbiome on even adjoining teeth(12). Hence, understanding the dynamics and structure of the peri-implant microbiome during *de novo* colonization will improve our ability to prevent, diagnose, and monitor peri-implant diseases.

Therefore, we aimed to investigate the sequence of bacterial colonization around dental implants in humans from initial exposure to the oral environment (uncovery) until after functional loading with the placement of a permanent crown by combining a longitudinal clinical study design with whole-genome shotgun sequencing, fine-scale enumeration and graph theoretics.

## RESULTS

15 subjects who gave informed consent and were initially enrolled completed all visits with no dropouts. Table 1 summarizes the demographic and clinical baseline information of the patients included. In summary, the study population included 8 females and 7 males; their average age of 59.53 (±14.13) years old. Twelve subjects were Caucasians, two were of Hispanic origin, and one identified as African-American. All implants installed were TSV with microtextured surface (Zimmer Biomet^®^, Palm Beach Gardens, FL), with 73.3% placed in the maxilla. No incidents of clinical or mechanical complications were identified through the study follow-up.

Whole genome shotgun sequencing generated 3.7 billion paired-end sequences (250bp). These sequences represented 17,777 functionally annotated microbial genes (based on KEGG classification), 548 species-level, and 2109 strain-level sequence identifications (based on eHOMD classification).

### The coverscrew chamber is the preponderant source of the peri-implant microbial community at 24 hours:

Over 500 species were identified in varying abundances in the coverscrew chamber, indicating that this environment hosts a rich and diverse community. *Streptococcus mitis* (19.123%), *Enterococcus casseliflavus* (7.20%), *Cutibacterium acnes* (5.58%), *Haemophilus parainfluenzae* (3.44%), and *Saccharibacteria (TM7) [G-1] bacterium HMT 348* (3.40%), were the most abundant species (Figure 2A and **Supplementary Table S1**). Of all 548 species identified, 240 were present in 100% of the coverscrew chambers (core microbiome), indicating that almost half (43.87%) of the microbiome is conserved among all implants at uncovery. Furthermore, these core species contributed to 78% of the microbial abundance in the coverscrew chamber, indicating that most of the heterogeneity in the coverscrew chamber microbiome is attributable to rare taxa.

When a Bayesian modeling approach was used to estimate the source of the 24-hour peri-implant microbiome, the coverscrew chamber was identified as the primary source, contributing 62.07 ± 14.73% of the bacterial species identified (p<0.05, Kruskal-Wallis). The adjoining teeth contributed 27.8 ± 10.30%, while 10 ± 10.70% of the peri-implant microbiome was traced to unknown environmental sources (potentially saliva or other oral mucosal surfaces, Figure 2B, Kruskal-Wallis, p<0.005).

### Coverscrew derived pioneer species drive the dynamics of peri-implant colonization:

We then interrogated the importance of these early colonizers on subsequent community dynamics using co-occurrence network analysis. Since the coverscrew chamber formed the predominant source of the 24-hour peri-implant microbiome, we designated the core coverscrew chamber bacteria (240 species) as “pioneer species”. Bacterial networks demonstrated progressively greater connectivity (with a 2-fold increase in the connections with increasingly larger hubs from uncovery to 42 weeks), indicating diversification of community synergy. Interestingly, even though the hubs expanded over time, they were anchored by the same consortia of species. Even more interesting was that most of these anchors were pioneer species (Figures 3A–F and **Supplementary Table S2**). While new species were introduced into the peri-implant microbiome beginning at 24hours, pioneer species dominated the nascent microbiome 70:30 throughout the observation period. A within-module/across-modules plot (ZiPi plot) analysis identified 15 species with a high degree of connectedness across different modules (Pi > 0.62). All 15 were pioneer species, and 13 of them belonged to the genus *Prevotella* (Figure 4A), further attesting to the influence of pioneers on network topography.

### New species are recruited to the microbiome based on phylogenetic relatedness to pre-existing colonizers:

Since the order of bacterial assembly has significant implications for colonization dynamics, we applied a mathematical model that quantifies the degree (D) to which new species inducted into a community are phylogenetically related to pre-existing community members. We discovered that implant colonization follows a significant phylogenetic underdispersion (D < 0, p = 0.0005), indicating that newly introduced species are more likely to be a phylogenetic relative of resident colonizers than random recruits (Figures 4B–C). We also discovered that the underdispersion pattern was not significant when the abundances of new or pre-existing species were inducted into the model (p > 0.05), indicating that the mere presence of a species is sufficient to promote colonization by a close phylogenetic relative, even if the species is present in low abundances.

We then examined the importance of pioneer species to the longitudinal acquisition of subsequent species using eLSA (Figure 4D, p<0.05, and **Supplementary Table S3**). 1890/3788 (50%) of edges connected pioneer species across the different time points. 128 edges were intra-generic, while 3660 were inter-generic, suggesting a process beyond phylogenic similarity in bacterial acquisition. Furthermore, 3414/3660 (93.28%) of the edges were identified at uncovery, and 67.63% of these were sustained over the entire timeline of the study; pointing to a sustained effect exerted by the pioneer species. Interestingly, 94 of the 128 edges demonstrated a negative association over time. For example, the densest intra-generic association was within the genus *Prevotella*, where 66 of the relationships were negative, while 11 were positive. The negative relationship began at uncovery and was sustained throughout the 12 weeks, indicating an early niche saturation that excludes same-genera species from the concomitant acquisition.

### The peri-implant microbiome demonstrates increasing personalization over time with the inclusion of new species:

Building on our previous investigations that implant surfaces exert a significantly greater selection pressure on the microbiome than host-associated factors(34), we calculated the level of homogeneity among subjects by computing the core microbiome at each time point at the species and sub-species/strain levels (Figure 5). In contrast to the time of uncovery when 49% of the species were shared among all 15 individuals, only 19–29% of the new recruits were shared among all implants in the subsequent time points, pointing to an increasing personalization of the peri-implant microbiome as it matures (**Supplementary Table S4**).

### The microbiome is functionally diverse across time and resilient to perturbation:

Since community development is driven by shared and competing functions within an ecosystem, we inducted 17,777 functionally annotated genes into alpha- and beta-diversity analyses. Compositional Tensor Factorization revealed significant expansion of functionalities from uncovery and each subsequent time point until the placement of the final restoration two weeks after uncovery, which served to reset the functionality to baseline levels at week 3 (p <0.0001 REML test, Figure 6A, CTF, Compositional Tensor Factorization). However, functional expansion renewed between 3 to 6 weeks and continued until 12-weeks.

At uncovery, the key functionality was transcription, and KRAB domain-containing zinc finger protein contributed predominantly to this function. Genes encoding pathways of oxidative phosphorylation (G-coupled receptor proteins, mono-oxidases, pyruvate metabolism etc.), fluid-exchange across membranes (aquaporins) were also predominant. Interestingly, at 24-hours, genes related to oxidative phosphorylation were decreased by 21%, indicating an increase in anaerobic functionality. This was also accompanied by a bloom in genes encoding replication, recombination and repair proteins, mitochondrial biogenesis, etc., consistent with community expansion through bacterial cell division (Figure 6B). When the 1-week biome was compared to the 24-hour biome, 25 genes encoding enzyme families (hydrolases and isomerases), 20 encoding membrane transport, cellular processes and signaling were enriched (Figure 6C, p < 0.05, FDR adjusted Wald test). On the other hand, 38 genes that coded for carbohydrate metabolism and enzyme families decreased in abundance (Figure 6D, p < 0.05, FDR-adjusted Wald test). No significant differential abundances were evident at 3 weeks and beyond.

### Implant colonization trajectories differ from those of adjoining teeth in diversity and extent of expansion:

We next compared the trajectories of functional expansion in the peri-implant and adjoining subgingival microbiomes. A similar functional volatility pattern was observed over time; however, the tooth, when compared to the implant, showed a greater functional diversity from uncovery until 12 weeks (p <0.016 REML test of CTF, Figure 7A). In corroboration, throughout all visits, a significantly different functional diversity was observed in teeth compared to implants (p < 0.0001, Jaccard and Bray-Curtis indices, Figures 7B and C, respectively). A very interesting observation was that there was an appreciable and significant decrease in functional volatility in teeth at week 3, similar to that of implants, even though the subgingival sulcus was not perturbed in the same fashion as the peri-implant crevice (i.e. placement of a restoration) (p <0.001 REML test of CTF, Figure 7A). This suggests that implant placement might impact the subgingival microbiome and deserves further investigation.

## DISCUSSION

Our knowledge of the early microbial colonization of the peri-implant sulcus is gleaned from studies that utilized culture-based methods, targeted microbiologic assays, and 16S rDNA sequencing(35–41). These studies surmised that the peri-implant sulcus is colonized within 30 minutes following implant placement(40), a complex flora can develop within 2 weeks(35,36), and that subgingival and peri-implant microbiota were similar(35,37,39). However, when we combined whole genome shotgun sequencing with multiple bioinformatics pipelines to longitudinally analyze the timeline for the acquisition, development, and personalization of the peri-implant microbiome from the moment the implant was uncovered through the following 12 weeks, we discovered an intricately orchestrated pattern of microbial assembly, in which the microbiota entrapped within the cover screw chamber play a key role.

Our principal finding was the importance of the coverscrew chamber microbiome in influencing peri-implant colonization. The first evidence was that the coverscrew chamber hosts a commensal-rich, diverse microbial community(42). While it is possible that these organsims were derived from saliva or other teeth, one notable observation was that the coverscrew chamber microbiome was homogeneous among all the subjects, suggesting that the implant material itseld might play an important role in modulating the flora, building a unique peri-implant niche. This lends credence to Becking and Beijerinck’s famous statement that “everything is everywhere, but the environment selects”(43). The second finding was the coverscrew chamber microbiota served as pioneer species. Pioneer species have adaptation abilities that help them colonize a habitat, and they grow fast(44–46). Evidence from non-human ecosystems shows that pioneers exploit the environmental changes that occur in newly created habitats (primary succession) or in recently disturbed environments (secondary succession) to influence community structure and composition(44,45). In line with this, we discovered that peri-implant pioneers exert an early and sustained effect as network influencers and controllers of resources. It was interesting to note that the peri-implant microbiome maintained a similar network topography in the face of progressive personalization and expansion; and that the hubs were predominantly anchored by the pioneers derived from the cover screw despite the “age” and idiosynchonization of the peri-implant community.

Another key finding was that bacterial succession in the peri-implant community follows an under-dispersion pattern of species recruitment. This implies that species are most likely to be recruited to this community if an evolutionarily close neighbor is already present(32,47). Interestingly, the mere presence of a ‘close neighbor’ is sufficient to promote colonization, suggesting that pioneer species can make the environment more favorable for a phylogenetic neighbor to colonize, irrespective of their abundance(47). This finding is particularly significant in patients with periodontitis, since pathogens can translocate from the affected tooth into the cover screw chamber during implant placement. This pathogen-rich community then seeds the pristine peri-implant sulcus, creating a virulent microbiome from inception. This provides a possible explanation for the higher risk of peri-implant diseases in subjects with untreated or poorly-controlled periodontitis.

In nature, bacterial succession is triggered by optimal ecological restoration, facilitative interactions, and subsequent competition(48–50). The accepted mechanism is that facilitative interactions occur between distantly-related species while competition occurs between closely-related species(48). Corroborating this, we observed robust and statistically significant negative correlations between members of the genus *Prevotella* throughout the colonization process. Within the implant community, we observed strong negative correlations between numerically dominant species with numerically rare taxa, and positive correations between numerically rare taxa.

In the present investigation, we discovered that shared functionality is an important determinant of bacterial succession; and that the functions encoded by the pioneers create an environment favorable for cell replication and biofilm maturation. Maturation is accompanied by a progressive increase in anaerobiosis from weeks 1–3. However, the predominant functionalities continue to be largely oxygen-dependent, namely, enzyme families, nucleotide metabolism, lipid metabolism, and energy metabolism. We also discovered that the community is functionally resilient since the dramatic decrease in functionality following the placement of a crown is rapidly restored to pre-restoration (visits before the placement of a crown) levels within the next 2 weeks. It is known that the functions expressed by members of a microbial community are impacted by their neighbors and by the physiological features of their environment(51,52); however, it was not within the scope of this study to investigate spatial relationships of the colonizers. Further studies that map the biogeography of the peri-implant sulcus are warranted to localize these interactions within the biofilm communities.

One final notable observation was that the colonization trajectories of implants differed from those of adjoining teeth, corroborating and building on our previous studies which have shown that the peri-implant biome is different from the tooth biome(12,53). Further, an interesting and significant influence was observed in the subgingival biome following the placement of an implant crown, suggesting that implant restoration might impact the subgingival microbiome, which deserves further investigation.

The present investigation used 15 subjects with a set of very stringent inclusion criteria. While this decreased the variability associated with clinical studies, it also decreased the generalizability of our data to other populations undergoing dental implants therapy (e.g., smokers, e-cigarette users, uncontrolled or poorly-controlled diabetics), as well as other implant systems, materials and surfaces. We also used a two-stage protocol; therefore, the findings cannnot be extrapolated to one-stage or one-piece implants. The two-stage protocol also limited our ability to track the sources of microbiota within the cover screw chamber at the time of implant placement. It is possible that saliva, the adjoining tooth, the adjacent subgingival crevice and the mucosa were all sources of the coverscrew bacteria at the time of implant placement. However, the implants were carefully isolated during second-stage surgery and cover screw retrieval.

In summary, within the study’s limits, we find evidence to support progressive personalization and expansion of the peri-implant microbiome over 12 weeks. Our data underscores the importance of pre-existing species in the oral environment during implant placement and highlights the importance of placing implants in periodontally healthy individuals or following the successful resolution of periodontal disease.

## MATERIAL AND METHODS

### Ethics statement

This was a single-center, longitudinal cohort study approved by The Ohio State Institutional Review Board (2013H0134) and carried out according to the approved protocol. The study conformed to the STROBE guidelines for observational studies. All participants gave written informed consent prior to enrollment, and the clinical study was carried out between June 2013 to September 2014. Samples were stored at −80°C, and analyzed in 2022 following the acquisition of further funding.

### Subject selection

We recruited systemically [ASA 1 (American Society of Anesthesiologists Physical Status Classification I)] and periodontally healthy individuals [attachment loss ≤ 1; less than three sites with 4 mm of probe depths (PD); bleeding index (BOP) ≤20%]. Inclusion criteria included partially edentulous individuals ≥ 21 years of age who were scheduled to receive a single, tooth-bounded tapered screw-vent (TSV) implant with microtextured surface as a part of a two-stage protocol in the Graduate Periodontology Clinics of The Ohio State University. Exclusion criteria consisted of being a current or former smoker (defined as those who had smoked more than 100 cigarettes in their lifetime) or e-cigarette user, controlled or uncontrolled diabetes, HIV infection, use of immunosuppressant medications, bisphosphonates or steroids, antibiotic therapy or oral prophylactic procedures within the preceding 3 months, requirement for antibiotic prophylaxis before dental examinations, and fewer than 20 teeth in the dentition.

#### Sample size estimation:

We based our sample size estimation on our peri-implant dual transcriptomic study(11), experimental gingivitis studies (13,14) and longitudinal transcriptional analysis of the periodontitis (15). In a typical representation of the peri-implant transcriptome, the transcripts are categorized by approximately 20 pathways. In our power calculations, we took a slightly conservative approach and assumed we would look at approximately 25 pathways in this data and will need to adjust for multiplicity using the Holm-Bonferroni method, which assures an experiment family-wise error rate of less than 5% if we use a critical value of 0.002 = 0.05/25 on individual analyses. By interrogating data from 15 subjects over 6-time intervals, we anticipated that we would be able to detect a contrast in the transformed scale of 1.59 degrees for the row (baseline versus each visit) effects.

### Study design

**Figure 1:**
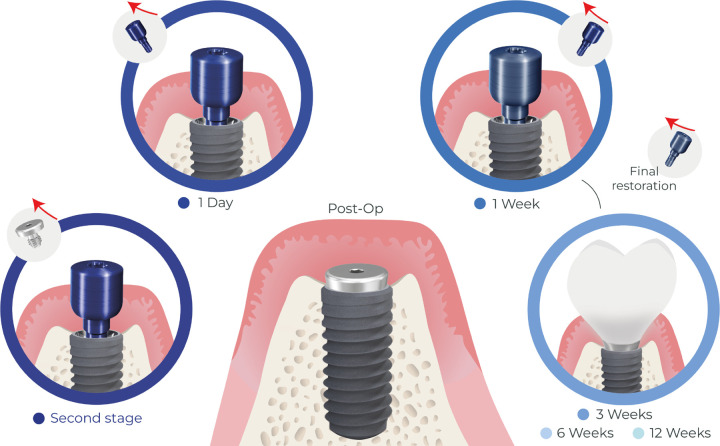
Clinical study design.

**Figure 2:**
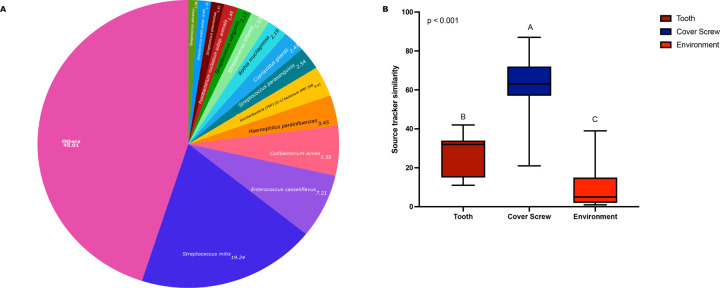
The coverscrew chamber as a source of pioneer species of the peri-implant microbiome Figure 2A represents species-level composition of coverscrew chamber microbiome at uncovery. Species present at a relative abundance of ≥1% are included in the pie chart. Taxa with an average relative abundance <1% are grouped in “Others”. Figure 2B is the sources of the pioneer species in the pristine implant sulcus. The implant and tooth at uncovery were set as the sources and implant at 24 hours as the sink (p<0.05, Kruskal-Wallis test). Boxplots not connected by same letter are significantly different.

**Figure 3:**
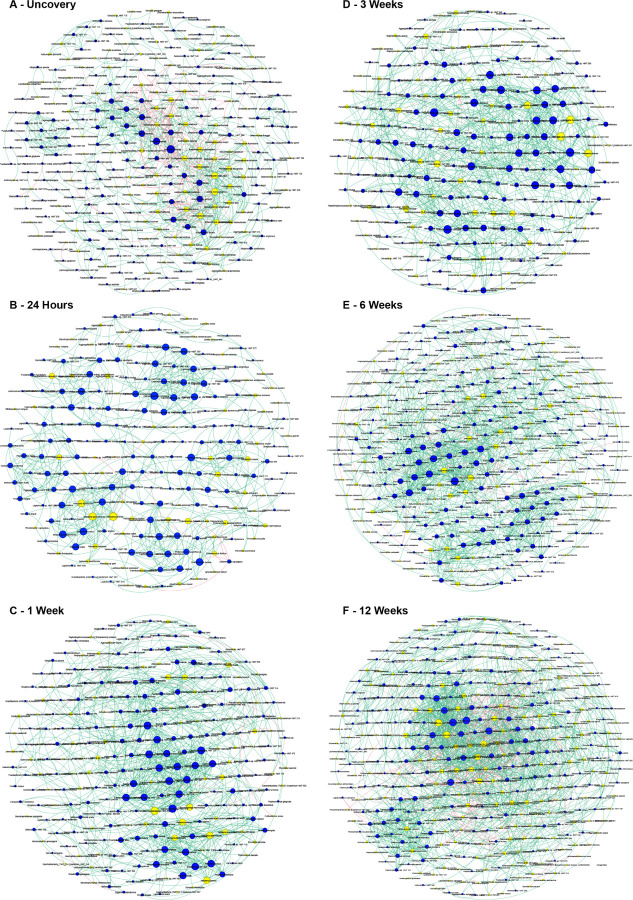
Community dynamics during the development of the peri-implant sulcus. Network graphs of implant at baseline, 24 hours, 1 week, 3 weeks, 6 weeks, and 12 weeks based on Sparse Co-occurrence analysis are shown in panels A-F. Each network graph contains nodes (circles sized by relative abundance per group) and edges (lines). Nodes colored in blue represent the pioneer species, while yellow nodes represent new species. Green edges represent positive correlation, while red edges represent negative correlation (r≥ |0.80|). Data supporting this figure can be found in Supplementary Material Table S2.

**Figure 4:**
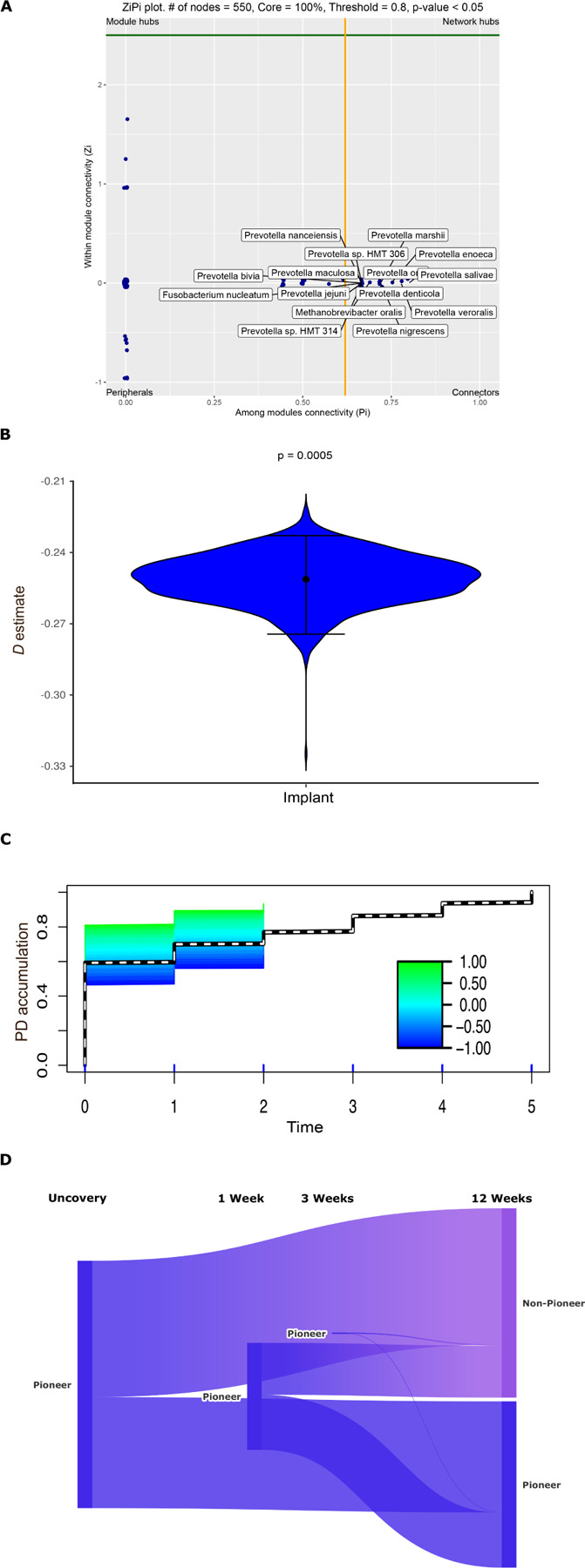
Recruitment of species follows principles of nepotism while pioneer species impact recruitment of new species and flow of resources. Within-module/across modules plot (ZiPi plot) analysis of the nodes following module identification with SCNIC(Pi > 0.62) are shown in Panel A. Panel B demonstrates empirical (dashed) and surrogate phylodiversity accumulation over the 12 week observation period. Surrogate curves are colored according to the dispersion (D) value. New species with a previously detected close relative are shown in blue, while those that do not have a close relative are shown in green. The surrogate curve (teal) is above the empirical curve (dashed), indicating that species recruitment is phylogenetically constrained, and follows nepotism. Panel C represents the dispersion parameter (D) estimates as a violin plot. The dot in the center of a violin is the mean, and bars represent 95% confidence intervals for the D estimate. Panel D represents a Sankey diagram of the Extended local similarity analysis (eLSA) revealing associations between pioneers and non-pioneer species over 12 weeks. Data supporting this figure can be found in Supplementary Material Table S3.

**Figure 5:**
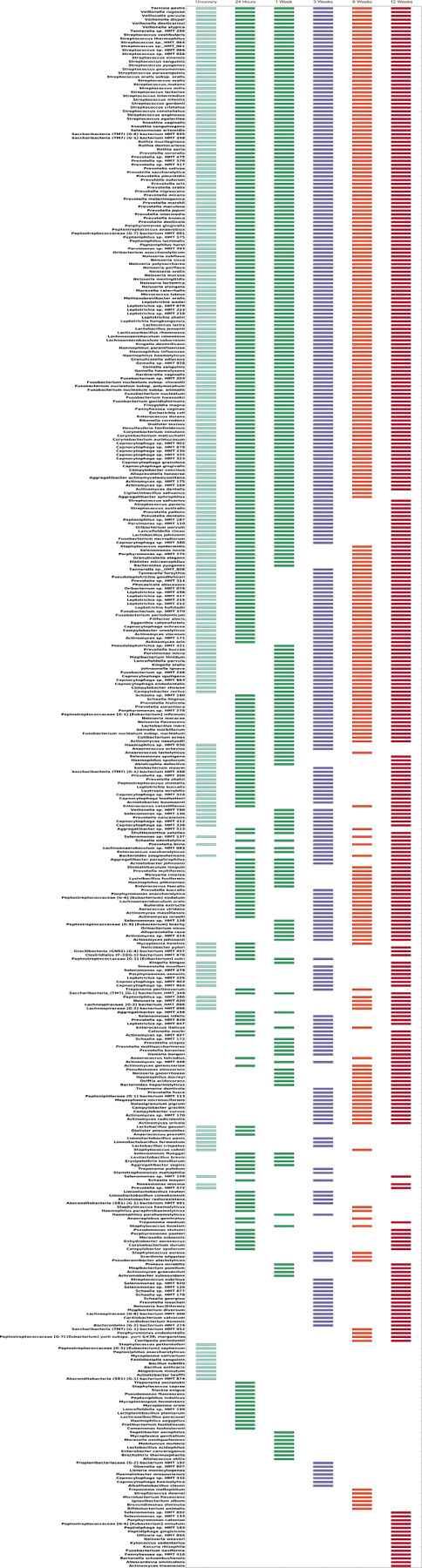
Is a waterfall plot of the core microbiome (species present in ≥100% of individuals) over time. Each bar indicates the presence of a species at the particular time point.

**Figure 6:**
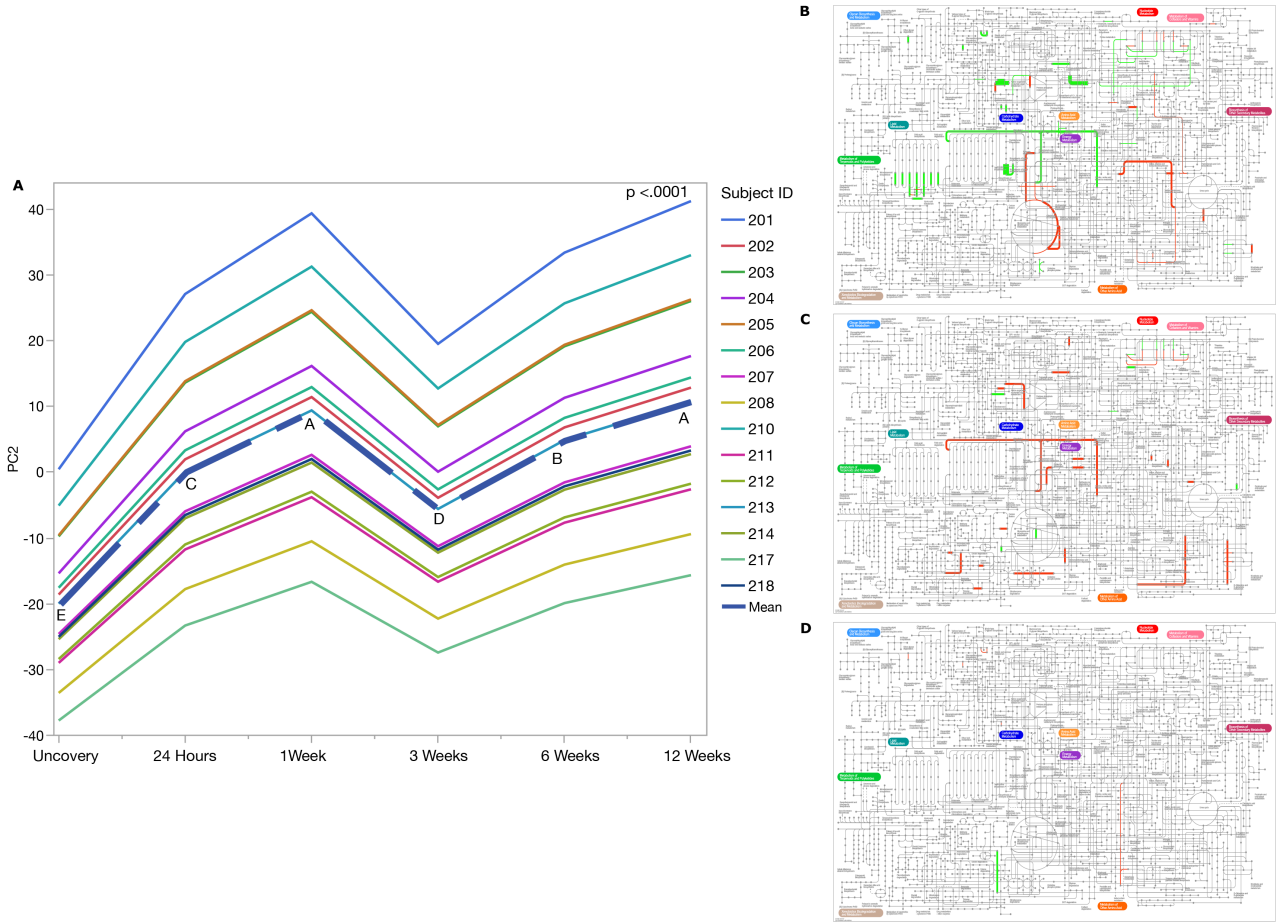
Functional dynamics of the developing peri-implant microbiome demonstrates stability after 3 weeks. Panel A shows b-diversity across time, as estimated by Compositional Tensor Factorization of functional genes. There was a significant increase in functional diversity between baseline and 3-weeks, followed by functional stabilization (p <0.0001 REML test). Panels B-D represent functional pathways that were diiferentially abundant during the 12-week observation period (P<0.05, FDR-adjusted Wald Test). Line thickness is sized by log⁡(2) fold change. Panel B: Differences between uncovery and 24 hours. Panel C: Differences between 24 hours and 1 week. Panel D: Differences between 1- and 3-weeks. No signifncat differences were detected after 3 weeks.

**Figure 7:**
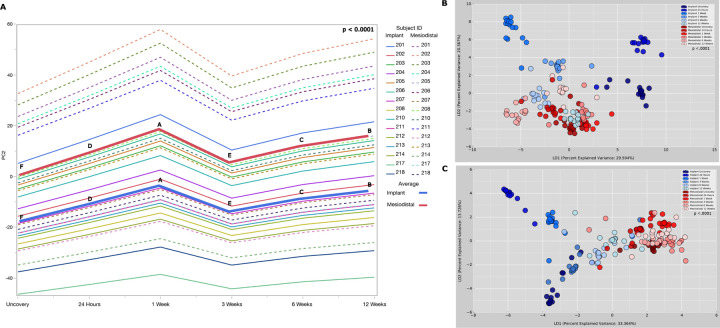
Implant colonization trajectories differ from those of adjoining teeth in diversity and extent of expansion. Panel A shows b-diversity across time, as estimated by Compositional Tensor Factorization of functional genes (Location: Implants versus Adjoining Teeth, p <0.016; Visits: p <0.0001; Visit*Location: p <0.0001, REML test). Panel B shows Linear discriminant analysis (LDA) of Jaccard index clustered by site (implant and teeth) and visit, The microbiome demonstrated significant differences based on both, site type and time (p <0.0001 REML test). Panel C shows Linear discriminant analysis (LDA) of Bray-Curtis distances clustered by site (implant and teeth) and visit. The microbiome demonstrated significant differences based on both, site type and time (p <0.0001 REML test).

Briefly, under a sterile and controlled surgical procedure and after extra- and intra-oral asepsis, the surgical area was anaesthetized, a modified crestal incision was made and a full thickness mucoperiosteal flap was raised. Implants were placed at bone level, and covered with a 0 mm healing abutment (also known as a cover screw), and the overlying mucosa was secured by primary closure (Figure 1). The implants were surgically uncovered 3 months later under isolation with sterile cotton swabs, and the cover screw was removed. Six sterile endodontic paper points (Caulk-Dentsply, Milford, DE, USA) were inserted into the cover screw chamber for 30 seconds and stored. The cover screw was replaced by a transmucosal healing abutment. Indices of periodontal health (gingival index –GI(16), plaque index – PI(17), and bleeding on probing - BOP) were recorded on the adjoining teeth. Baseline biofilm samples were collected from the adjoining teeth by inserting six paper points into the gingival sulcus of adjoining teeth. The adjoining teeth were scaled, polished, and flossed to remove adherent biofilm and monitor de novo colonization of the teeth’s subgingival microbiome simultaneously with the implant. Following this baseline visit, the participants were seen for 5 more visits (24 hours, 1 week, 3 weeks, 6 weeks, and 12 weeks). At each visit, the healing abutment was removed and collected, and paper points were inserted into the developing peri-implant crevice and stored along with the healing abutment. A new abutment was placed at the end of each visit. Subjects were seen for placement of a final restoration after two weeks, following which the protocol continued in a similar fashion.

#### Sample collection:

Prior to sample collection, selected sites were isolated with cotton rolls, and supragingival plaque was removed. Subgingival and submucosal biofilm samples were collected from each patient every visit by inserting six sterile endodontic paper points into the peri-implant and the adjoining periodontal sulci for 30 seconds. The samples were then separately placed in two 1.5 ml microcentrifuge tubes containing 100μL of RNA*Later*. Samples were frozen at 80°C until further analysis.

### DNA Isolation and Sequencing

Bacteria were separated from the paper points by adding 200ul of phosphate-buffered saline to the tubes and vortexing. The paper points were then removed, and DNA was isolated with a Qiagen DNA MiniAmp kit (Qiagen, Valencia, CA, USA), following the protocol according to the manufacturer’s instructions. Libraries were generated using the NEBNext^®^ UltraTM II FS DNA Library Preparation Kit for Illumina (New England Biolabs, Inc). 100 ng of the sample was used as input. DNA was fragmented to 100 – 250 bp. Adapters were then ligated to the fragments. 12 unique adapters were used so that 12 samples could be pooled together during sequencing. Size selection was not performed; however, the adapter-ligated samples were cleaned before PCR enrichment. 6 cycles of PCR were completed before cleaning and pooling the samples. Pooled libraries were sequenced on the Illumina HiSeq4000 platform using 150bp paired-end chemistry.

### Metagenomic Data Processing and Statistical Analysis

**Metagenomic sequence analysis:** Sequences were quality filtered with Sickle and screened for human DNA with(18). Phylogenetic assignment was performed using Woltka and Bowtie 2 mapping to the Human Oral Microbiome Database (HOMD)(19). Prodigal was used for coding sequence (CDS) prediction, and genes were aligned against the NCBI nonredundant database of proteins using DIAMOND(20). Biological pathways and protein functional categories were determined by annotation to the Kyoto Encyclopedia of Genes and Genomes orthology (KEGG)(21) using MEGAN(22). **Bioinformatic analysis** was performed using the QIIME 2 2023.5(23) and PhyloToAST v1.4(24). Alpha; alpha diversity was evaluated through calculation of the Shannon index(25) and significance of longitudinal differences through repeated measures ANOVA. Longitudinal estimation of beta diversity differences using the Jaccard and Bray-Curtis indices was computed using Compositional Tensor Factorization as part of the TEMPoral TEnsor Decomposition (TEMPTED) analysis framework(26,27). Linear Discriminant Analysis was used for dimensionality reduction and ordination. The bayesian analysis-based SourceTracker(28) was used to identify probable sources of the peri-implant sulcus microbiome.

Colonization dynamics over time were ere examined using (1) Network analysis: Sparce Co-occurrence Network Investigation for Compositional Data (SparCC) to compute species-level co-occurrence networks for each time point (r>0.80). A correlation network was built, and modules were detected using SCNIC(29). Network graphs were visualized in Gephi (30), and within-module/across-modules analysis was performed using the built-in tools. (2) Extended Local Similarity Analysis (eLSA): was used to identify the longitudinal effect of the species on the acquisition of subsequent species, which examines the co-occurrence (edge) of species X on Species Y across different time points, while reporting both the strength of the relationship, and the timepoint when Species X started affecting species Y, and whether there is a delay in this effect (31). (3) Phylogenetic model for species recruitment: This model, described by Darcy et al. (32), tests whether new species are more or less likely to be recruited into the community in the presence of a close phylogenetic relative (nepotism) or if the process was agnostic to evolutionary relationships (neutral model). The model estimates an empirical dispersion parameter “*D*”, which quantifies the degree to which first-time species are phylogenetically related to the pre-existing community residents. If D ≠ 0, then species are preferentially added if they have relatively close (D < 0) or relatively far (D > 0) phylogenetic distance to the resident community,

Differential abundance analysis of functional genes was carried out using DESeq2 and p-values adjusted for multiple testing (false discovery rate [FDR]<0.05, FDR-adjusted Wald test) (33). The results were used to construct KEGG maps using the web-based Interactive Pathways Explorer (iPath3) (33). Statistical analysis for longitudinal and within-site comparisons was conducted using JMP software using the restricted maximum likelihood method (REML) to reduce biases in variance and co-variance.

Sequences are deposited in the NCBI Sequence Read Archive under the project ID PRJNA1082341.

## Figures and Tables

**Table 1.** Baseline sociodemographic and clinical characteristics of the study population.

	No. of patients (%) or parameter value
*Age (Years ± SD)*	59.53 ± 14.13
*Gender (%F)*	53.55%
*Race – (%Caucasion)*	80%
*Implant Location - (%upper-arch)*	73.3%
*Plaque index (mean ± SD)*	0.03 ± 0.12
*Gingival index (mean ± SD)*	1.43 ± 0.86
*Bleeding on probing (%Yes)*	93%

## Data Availability

the sequence materials have been uploaded to the Sequence Read Archives (SRA) of the National Center for Biotechnology Information (NCBI) database with the following identifier: PRJNA1082341.
